# A transcriptional reference map of defence hormone responses in potato

**DOI:** 10.1038/srep15229

**Published:** 2015-10-19

**Authors:** Lea Wiesel, Jayne L. Davis, Linda Milne, Vanesa Redondo Fernandez, Miriam B. Herold, Jill Middlefell Williams, Jenny Morris, Pete E. Hedley, Brian Harrower, Adrian C. Newton, Paul R. J. Birch, Eleanor M. Gilroy, Ingo Hein

**Affiliations:** 1 The James Hutton Institute, Invergowrie, Dundee, DD2 5DA, UK; 2 The Division of Plant Sciences, College of Life Science, University of Dundee at the James Hutton Institute, Dundee, UK

## Abstract

Phytohormones are involved in diverse aspects of plant life including the regulation of plant growth, development and reproduction, as well as governing biotic and abiotic stress responses. We have generated a comprehensive transcriptional reference map of the early potato responses to exogenous application of the defence hormones abscisic acid, brassinolides (applied as epibrassinolide), ethylene (applied as the ethylene precursor aminocyclopropanecarboxylic acid), salicylic acid and jasmonic acid (applied as methyl jasmonate). Of the 39000 predicted genes on the microarray, a total of 2677 and 2473 genes were significantly differentially expressed at 1 h and 6 h after hormone treatment, respectively. Specific marker genes newly identified for the early hormone responses in potato include: a homeodomain 20 transcription factor (DMG400000248) for abscisic acid; a SAUR gene (DMG400016561) induced in epibrassinolide treated plants; an osmotin gene (DMG400003057) specifically enhanced by aminocyclopropanecarboxylic acid; a gene weakly similar to *AtWRKY40* (DMG402007388) that was induced by salicylic acid; and a jasmonate ZIM-domain protein 1 (DMG400002930) which was specifically activated by methyl jasmonate. An online database has been set up to query the expression patterns of potato genes represented on the microarray that can also incorporate future microarray or RNAseq-based expression studies.

Potato is the third most important food crop in the world and sustainable production is currently dependent on the application of chemicals or the development of new cultivars to achieve tolerance or resistance to biotic and abiotic stresses^1^. The major pathogens of potato include the oomycete *Phytophthora infestans*, cause of late blight, Potato Cyst Nematodes (PCN), viruses such as *potato virus Y* (PVY) and bacteria including *Ralstonia solanacearum* (1 and references within). Abiotic pressures that impact on yield stability comprise, amongst others, heat, cold and water stresses^2^,^3^,^4^.

Phytohormones regulate diverse aspects of plant life^5^,^6^. For example, the hormones brassinosteroids (BR) including the most bioactive form brassinolide (BL), and abscisic acid (ABA) are predominantly involved with plant growth and development but can, as shown for BL, also feature in defence responses^7^,^8^,^9^,^10^. Indeed, phytohormones play important roles in regulation of stress and defence responses^8^,^11^. Salicylic acid (SA), ethylene (ET), jasmonic acid (JA), and its derivatives (called jasmonates), are typically associated with the regulation of biotic stress responses^12^,^13^. The SA pathway is associated with responses to biotrophic pathogens, whereas JA and ET, which are generally thought to act synergistically, are linked to defences against necrotrophic pathogens and herbivorous insects^12^,^14^.

Phytohormone signalling is conveyed through a complex network with considerable cross-talk to facilitate the required fine-tuning and modulation of responses to environmental cues^5^. The majority of hormone studies have been conducted in the model plant *Arabidopsis thaliana* and are based on mutants that are impaired in the biosynthesis of hormones or downstream signalling cascades. Recent studies on crops like rice suggest that responses can vary between species and unique complexities in defence regulatory networks have been revealed^13^,^15^,^16^,^17^. Moreover, compared to *A. thaliana*, both rice and potato have, for example, elevated basal levels of SA^18^. Although the impact of the different basal hormone levels remains elusive, it highlights the need for species-specific hormone studies.

The sequencing of the potato genome^19^ has enabled the design of a gene expression microarray that encompasses a representative probe set for transcripts of the predicted gene models^20^. The aim of this study was to generate a transcriptional reference map of the early potato responses to exogenous application of five major plant hormones ABA, BL (applied as epibrassinolide [Epi]), ET (applied as aminocyclopropanecarboxylic acid [ACC], a precursor in ET biosynthesis), JA (applied as methyl jasmonate [Me-JA]) and SA, and to identify potential marker genes for the individual hormone pathways in potato. An online database to query the expression patterns of potato genes represented on the microarray has been setup at http://ics.hutton.ac.uk/solarray.

## Results

### Overview of genes affected by the different plant hormones

**Figure 1:**
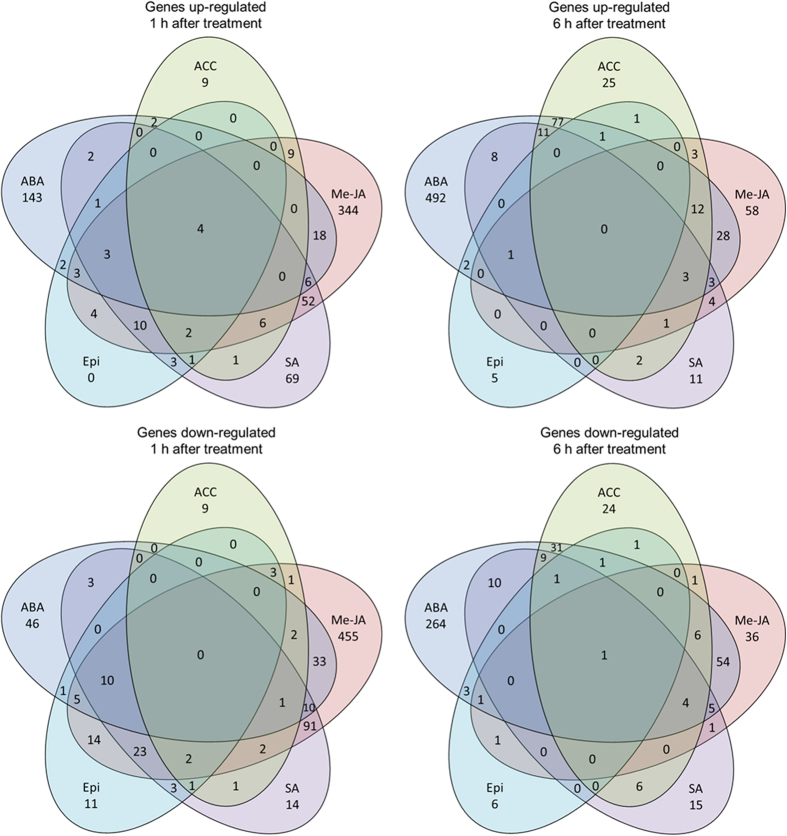
Numbers of genes significantly affected in their expression in hormone treated vs. control plants (p-value ≤ 0.05). Numbers of genes uniquely affected by one of the hormones and numbers of genes affected by more than one hormone are shown. Only genes that were consistently up- or down-regulated across the treatments were included in this figure. Genes that were effected in opposite direction (e.g. up-regulated by ABA but down-regulated by ACC) are not included. The figure displays genes up-regulated 1 h after treatment, genes down-regulated 1 h after treatment, genes up-regulated 6 h after treatment and genes down-regulated 6 h after treatment. ABA (abscisic acid); ACC (aminocyclopropanecarboxylic acid); Epi (epibrassinolide); Me-JA (methyl jasmonate); SA (salicylic acid).

All five plant hormones had considerable effects on plant gene expression both at 1 h and 6 h after application. A total of 2677 genes showed significantly differential transcript abundance at 1 h after any of the hormone treatments and a total of 2473 genes at 6 h (p-value ≤ 0.05) (Table S1). At both time points, overlap in gene expression was observed, but large numbers of genes were uniquely up- or down-regulated by only one of the hormones (Fig. 1). Genes that were, dependent on the hormone treatment, induced or suppressed in their transcript abundance (referred to as affected in opposite direction) are highlighted in Table S1. The greatest influence on gene expression at 1 h after treatment was the application of Me-JA. In total, 344 genes were uniquely induced by Me-JA and 455 genes were uniquely down-regulated. Genes encoding members of the cytochrome P450 family were the highest (up to 25-fold) uniquely up-regulated group in Me-JA treated plants at 1 h after treatment (DMG400001098, DMG400001942, DMG400001944, DMG400015183, DMG400015184, DMG400015185, DMG400015242, DMG400016776, DMG401001941, DMG401010026, DMG402001903, DMG402001941). Genes involved in the JA biosynthesis and signalling pathways were also up-regulated 1 h after Me-JA application. These included a gene for lipoxygenase (DMG400022894) that was 2-fold induced compared to the water control and a JA-induced WRKY transcription factor (DMG400019824) that was 4-fold up-regulated. Genes for heat-shock proteins were the most (up to 13-fold) uniquely down-regulated group of transcripts in Me-JA treated plants at 1 h post treatment (DMG400000444, DMG400000398, DMG400005573, DMG400006271, DMG400009509, DMG400030405). Other genes uniquely down-regulated by Me-JA application encode EIX receptors (DMG400022978, DMG400022988, DMG400022990; 2-6-fold down-regulated) and ATP binding proteins (DMG400012203, DMG400015170, DMG400019514, DMG400030572, DMG400031081, DMG400044751, DMG401019239, DMG402003983, DMG402008483; 2-5-fold down-regulated).

At 1 hour post treatment, ABA, ACC and SA uniquely induced 143, 9 and 69 genes and specifically suppressed 46, 9 and 14 genes, respectively. Conversely, Epi did not up-regulate any genes uniquely but specifically down-regulated 11 genes. Me-JA and SA showed the greatest overlap at 1 h post treatment with 52 genes induced and 91 down-regulated by both hormone treatments. Examples include a transcript for a multi-drug resistance protein (DMG400024532) that was 5-fold up-regulated by Me-JA treatment and 8-fold induced by SA treatment, and an ABC transporter family protein for which the gene (DMG400009095) was induced 5-fold and 12-fold, respectively. Genes involved in heat stress were strongly down-regulated by both Me-JA and SA applications and include, for example, chloroplast small heat shock protein genes (DMG400011628, DMG400011630, DMG400038479) that were 5-20-fold down-regulated by both treatments. At 1 h after treatment, four genes were significantly up-regulated by all five hormone treatments (p-value ≤ 0.05). One of the genes encoded a beta-galactosidase (DMG400030954) which was induced 6-fold by ABA, 4-fold by ACC, 8-fold upon treatment with Epi, 17-fold by Me-JA and 9-fold by SA.

The greatest influence on transcript abundance at 6 h after treatment was the application of ABA. In total, 492 genes were uniquely induced and 264 genes were specifically down-regulated by ABA. Genes involved in the ABA pathway in plants were strongly up-regulated and include, for example, the ABA and environmental stress-inducible protein TAS14 (DMG400003530) that was up-regulated 1720-fold. An MLO1 gene (DMG400020605) involved in stress response was 113-fold up-regulated by ABA. Furthermore, non-specific lipid transfer protein genes (DMG400011951, DMG400011952, DMG400011953, DMG400011954, DMG400012837, DMG400012838, DMG400012839, DMG400016102, DMG400020131, DMG401031237) were 5-165-fold up-regulated and protein phosphatase genes (DMG400002573, DMG400009112, DMG400016742, DMG400019604, DMG400020517, DMG400027196, DMG400030332, DMG400030410) were 5-40-fold up-regulated.

Whilst fewer genes were affected by Me-JA and SA applications at 6 h after treatment if compared to 1 h, ACC had a greater effect on gene expression at 6 h than at 1 h (Fig. 1; Table S1). At this time point, ACC, Epi, Me-JA and SA uniquely up-regulated 25, 5, 58 and 11 genes and down-regulated 24, 6, 36 and 15 genes, respectively. ABA and ACC showed the greatest overlap in gene induction at 6 h after treatment and affected 77 genes jointly. Among the proteins encoded by these genes were, for example, a WRKY-type DNA binding protein (DMG400021895) that was 17-fold up-regulated by ABA and 11-fold induced by ACC as well as a tryptophan decarboxylase (DMG400018358) that was 33-fold up-regulated by both ABA and ACC.

### Gene ontology classification of transcripts altered by plant hormones

**Figure 2:**
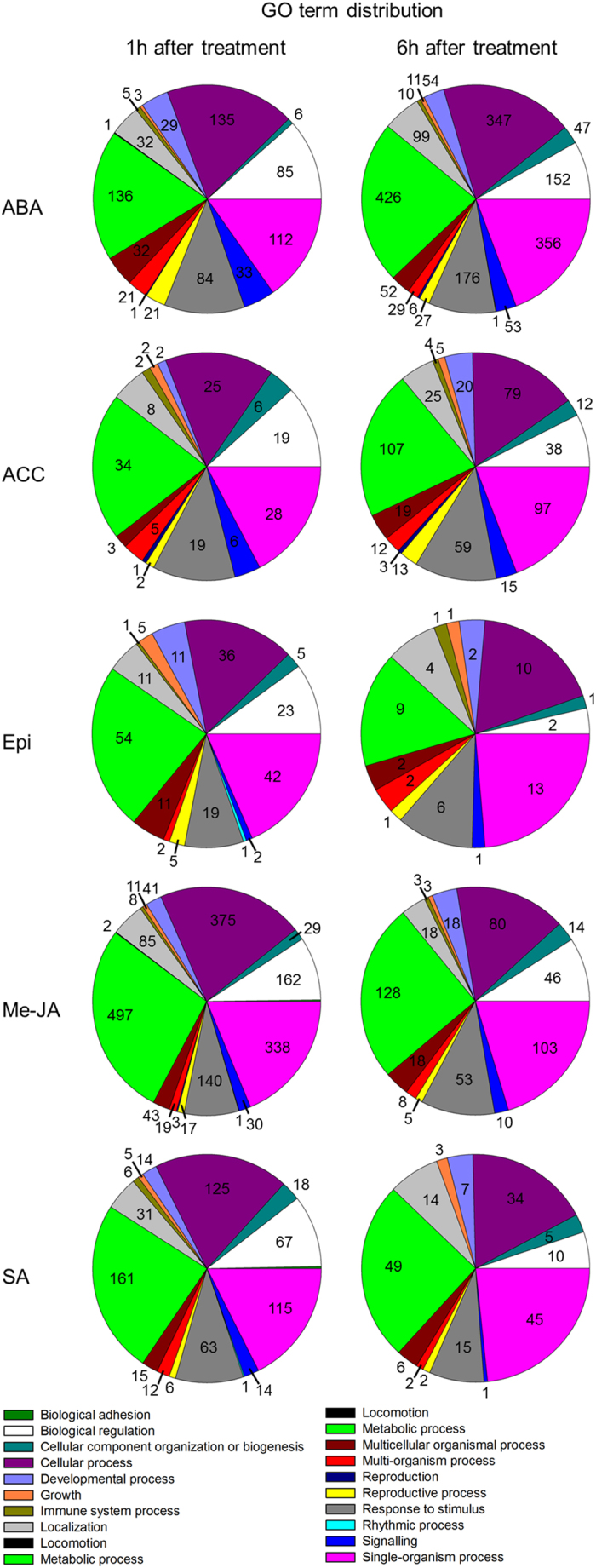
Gene ontology classification of biological processes affected by any of the five hormone treatments at 1 h and 6 h are shown. ABA (abscisic acid); ACC (aminocyclopropanecarboxylic acid); Epi (epibrassinolide); Me-JA (methyl jasmonate); SA (salicylic acid).

A GO classification was performed to identify biological processes that are influenced by treatments with the plant hormones (Fig. 2). Generally, genes involved in the GO term annotations ‘cellular processes’, ‘single-organism processes’ and ‘metabolic processes’ were altered the most by hormone treatments.

A GO term classification that was strongly influenced by all hormone treatments was ‘response to stimulus’. At 1 h after treatment, genes encoding for extracellular ligand-gated ion channels (DMG400013264, DMG400022304) were 3-fold up-regulated in ABA treated plants whereas SAUR family proteins (DMG400001614, DMG400001615, DMG400001667, DMG400001668) were 2-3-fold down-regulated by ABA and Me-JA. ACC treatment after 1 h led to 2-fold suppression of a multicystatin gene (DMG400005950). A peroxidase transcript (DMG401025083) was induced 5-fold by ACC, 2-fold by Epi, 9-fold by Me-JA and 3-fold upon SA treatment. Expression of oxidoreductase genes (DMG400011098, DMG400011929, DMG400019119) was 4-fold suppressed by Me-JA. At 1 h after treatment, SA strongly up-regulated UDP-glucose/glucosyltransferase genes (DMG400015579; 155-fold up-regulated and DMG400015601; 100-fold up-regulated).

Different gene families implicated in the GO term annotation ‘signalling’ were altered in transcript abundance by the hormone treatments at 1 h and 6 h. After 1 h, CBL-interacting protein kinase transcripts (DMG400028479, DMG400028491) were 3-fold up-regulated by ABA and ACC treatments, and 3-fold down-regulated by Me-JA. Me-JA application after 1 h also affected MAP kinase genes (DMG400007058, DMG400003528), which were 2-4-fold up-regulated. At 1 h and 6 h post-treatment, a total of 74 and 42 differentially transcribed transcription factor genes embedded in GO terms such as ‘signalling’, ’response to stimulus’, and ‘response to hormone’, were altered in their transcription, respectively (Table 1a,b). Seven of these genes were affected in opposite direction by the different hormone treatments at 1 h (Table 1a). AP2 domain-containing transcription factor 9 transcript (DMG400012154) was up-regulated by ABA and down-regulated by Me-JA whereas an AP2/ERF domain-containing transcription factor gene (DMG400008734) was up-regulated by SA and down-regulated by ABA. A gene encoding a COL domain class transcription factor (DMG400003711) was induced by Me-JA and supressed by ABA treatment. Transcripts for a MYB transcription factor (DMG400011250) and a MYB domain class transcription factor (DMG400019535) were strongly up-regulated in ABA treated leaves and down-regulated by Me-JA. Two WRKY transcription factor genes (DMG400001434, DMG400029207) were up-regulated by SA treatment and down-regulated in Me-JA treated potato leaves. At 6 h after treatment, only one gene was induced or suppressed by the different hormone treatments (Table 1b). This gene, encoding a COL domain class transcription factor (DMG400003711), was induced by Me-JA but repressed by ABA and these effects had also been seen at 1 h after treatment.

### Database to query the expression patterns of potato genes represented on the microarray

The microarray signals (both raw and normalised) are stored in a database alongside information concerning the corresponding probes and the PGSC genes (DMGs) and transcripts (DMTs). The database is searchable through the associated website http://ics.hutton.ac.uk/solarray. The website allows users to access the information by searching for sequence IDs (PGSC transcript/gene, or probe name), by conducting a BLAST search of a nucleotide or protein sequence against the PGSC transcripts to identify homologous sequences, or via annotation-based keyword search (Figure S1). The microarray signals are displayed in both graphical and tabular format for each probe and significant fold changes (p-value ≤ 0.05) are highlighted (Figure S1). The database is linked to the Spud DB Genome Browser (http://potato.plantbiology.msu.edu/cgi-bin/gbrowse/potato/) to facilitate additional analysis.

### Marker gene analysis

The closest homologs in potato of some previously described *A. thaliana* genes involved in hormone signalling or described as phytohormone markers were identified following a search for the reciprocal best hits or, if not discernible, the top potato protein blast hits and assessed for their gene expression profiles in potato (Table S2). The ET marker genes ETHYLENE INSENSITIVE 3 (AtEIN3) and ETHYLENE RESPONSE FACTOR 1 (AtER1) show closest similarities in potato to genes annotated as EIL1 (DMG400029908) and EFR10 (DMG400013402), respectively. The BR marker genes DWARF 4 (AtDWF4), CONSTITUTIVE PHOTOMORPHOGENIC DWARF (AtCPD) and EXPANSIN 8 (AtEXP8) are homologues in potato to cytochrome P450 genes (DMG400014902 and DMG400023819) and an expansin (DMG400016650), respectively. Genes involved in BR signalling BRASSINOSTEROID INSENSITIVE 1 (AtBRI1) and BRI1-ASSOCIATED KINASE 1 (AtBAK1) are homologous in potato to a BRI1 protein (DMG400019698) and the somatic embryogenesis receptor kinase 3B (DMG400012594), respectively. The JA marker genes encoding VEGETATIVE STORAGE PROTEIN 2 (AtVSP2) and PLANT DEFENSIN 1.2 (AtPDF1.2) show closest similarities to a glycoprotein (DMG400016494) and a defensin (DMG400008516), respectively. The SA marker genes PHENYLALANIN AMMONIA-LYASE (AtPAL1), PATHOGENESIS RELATED PROTEIN 1 (AtPR1) and NON-EXPRESSOR OF PR GENE 1 (AtNPR1) are homologous to genes annotated as a phenylalanine ammonia-lyase (DMG400031457), the basic PR-1 protein (DMG400005112) and PR1/NIM1 like defence protein (DMG401000923) in potato, respectively. All these genes were expressed in our experiments (Table S2), albeit the raw values of the homologs to ERF1, EXP8, PDF1.2, PR1 and NPR1 were below 10 and thus below the confidence threshold for detection. These genes were not differentially affected by any of the hormone treatments compared to the control (Table S2). Thus, none of these homologs of hormone-responsive marker genes in *A. thaliana* showed similar behaviour in potato. We therefore used the data presented here to develop marker genes for early, hormone-specific responses in potato.

### Validation of expression patterns using quantitative RT-PCR

**Figure 3:**
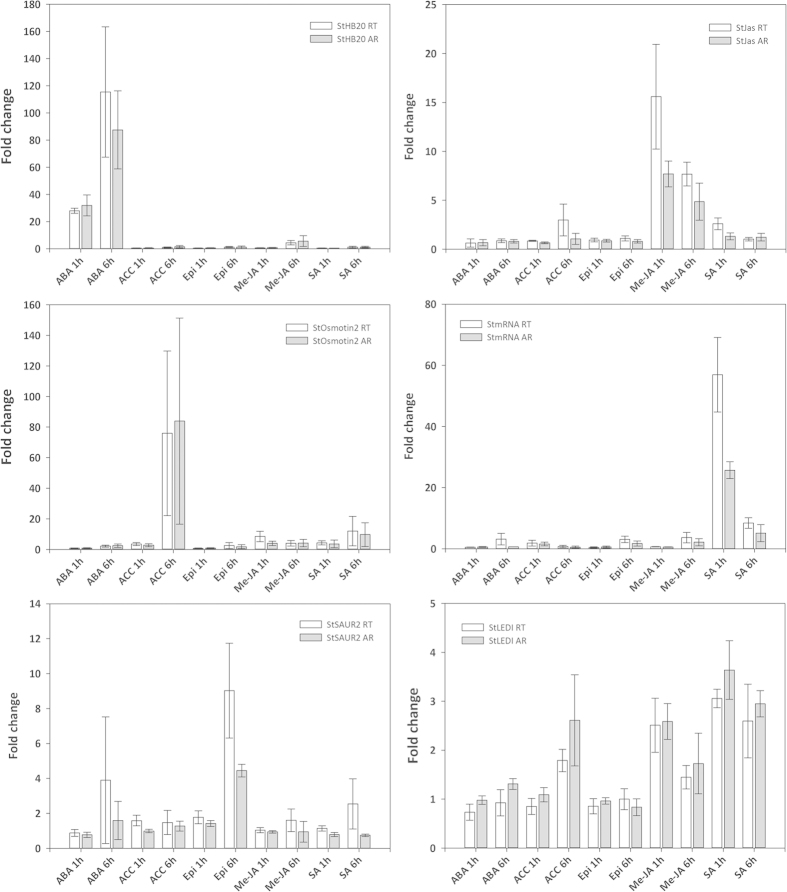
Fold changes in gene expression for six genes (DMG400000248 (StHB20), DMG400003057 (StOsmotin2), DMG400016561 (StSAUR2), DMG400002930 (StJas), DMG402007388 (StmRNA), DMG402018777 (StLEDI)) in response to treatment with hormones as measured by qRT-PCR (white) and microarray (grey) technologies are shown. Displayed are mean values (N = 3) and standard errors. ABA (abscisic acid); ACC (aminocyclopropanecarboxylic acid); Epi (epibrassinolide); Me-JA (methyl jasmonate); SA (salicylic acid).

To identify potato phytohormone markers suitable for the early time points used in this study, genes that displayed hormone-specific expression profiles were selected for further analysis (Fig. 1). Genes that were specifically induced by only one hormone, either at both 1 h and 6 h post treatment or at just one of the time points, were sought. Genes that belong to large families of closely related sequences were avoided and priority was given to genes that displayed large expression changes upon hormone treatment. Quantitative RT-PCR (qRT-PCR) was used to validate the microarray-based expression patterns of these selected genes (Fig. 3). One gene, encoding a LEDI-5c protein (DMG402018777) and which was influenced in its expression by Me-JA at 1 h and SA at 1 and 6 h, was included to verify more complex expression patterns as seen by microarray analysis (Fig. 3). In all cases, qRT-PCR corroborated the expression patterns observed in the microarray analyses. The ABA specific gene Homeodomain 20 transcription factor (DMG400000248; previously annotated as HB1 [Table S1]) displayed approximately 30-fold induction at 1 h after ABA treatment in both the microarray and qRT-PCR study and about 80-fold and 110-fold induction at 6 h in the microarray and the qRT-PCR analysis, respectively. An osmotin gene (DMG400003057), specifically induced by ACC, was around 80-fold up-regulated at 6 h post treatment on both platforms. An auxin-induced SAUR gene (DMG400016561) was significantly higher expressed in Epi treated plants at 6 h than in any other hormone treated plant (p-value ≤ 0.05). The jasmonate ZIM-domain protein 1 (DMG400002930) displayed specific transcript induction by Me-JA and was around 7-fold and 15-fold induced in microarray and qRT-PCR-based analysis at 1 h and 4-fold and 7-fold up-regulated at 6 h, respectively. As measured by microarray and qRT-PCR, a gene annotated to be weakly similar to *AtWRKY40* (mRNA, DMG402007388) was specifically induced by SA and displayed approximately 25-fold and 55-fold induction at 1 h after treatment with SA only.

## Discussion

Potato yields are affected by a variety of abiotic and biotic stresses and current research is aimed at finding new approaches to alleviate the impact of these threats^1^. The importance of integrated disease management to reduce biotic stresses on crop plants is rising and the application of elicitor compounds that enhance plant immunity is one strategy to eventually reduce the need for chemical pesticides^21^. The molecular mode-of-action of many elicitor compounds is, however, not fully understood but it has been shown that plant hormone pathways are often stimulated^20^,^22^,^23^,^24^,^25^. Since elicitors impact on gene expression mostly in the first few hours after application^22^, we have generated a reference map for the early changes of hormone-dependent gene regulation in potato following exogenous applications of abscisic acid (ABA), aminocyclopropanecarboxylic acid (ACC) a precursor in ethylene synthesis, epibrassinolide (Epi), methyl jasmonate (Me-JA) and salicylic acid (SA). *A. thaliana*, the most studied model plant, is of limited use for hormone studies in unrelated species since it has been shown, for example in potato and rice, that marked differences can exist in pathways and responses to hormones in major crop plants^16^,^17^,^20^.

Generally, more genes were influenced in their expression at 1 h than at 6 h after treatment, showing that in potato, responses to external applications of plant hormones are very rapid and therefore in agreement with previous studies^15^,^26^. However, each plant hormone differed in their temporal influences. The strongest response in potato at 1 h after treatment was alteration by Me-JA. SA also differentially regulated more genes at 1 h than at 6 h, but fewer genes were altered than by Me-JA. In contrast, ABA and ACC impacted most on gene expression at 6 h after hormone application. Most genes were uniquely affected by a single phytohormone and relatively few were affected by more than one treatment. This is in agreement with a previous study in *A. thaliana* that focused on the effects of hormones involved in growth regulation and concluded that hormones, if applied individually and in isolation, mostly affect gene expression independently^26^.

However, upon perception of environmental cues, phytohormone signalling pathways are strongly interconnected to allow plants to quickly and efficiently respond to biotic and abiotic stresses^5^. The crosstalk between different plant hormones has been well documented in *A. thaliana*^5^,^14^,^27^. The best-known examples are reported crosstalk between the defence hormones JA and SA. For example, in *A. thaliana*, the JA-responsive genes *PDF1.2* and *VSP2* are very sensitive to suppression by SA and the transcription factors WRKY50 and WRKY51 play roles in SA-induced suppression of the JA pathway^5^,^14^. However, parallel effects of hormone treatments have also been reported^5^,^12^,^27^ and have also been observed in this study. For example, 52 genes were up-regulated by both Me-JA and SA at 1 h after treatment and 91 were down-regulated. The transcript abundance of only 9 genes was affected in opposite direction. This equates to 94% of the genes being similarly affected and 6% in opposite directions. Similar results have been observed in rice, where 88% of genes were similarly affected by JA and SA^15^. In addition, in our potato study, 77 genes were up-regulated by both ABA and ACC at 6 h after treatment and 31 were down-regulated by both hormones at this time point. Only 7 genes were affected in the opposite direction. Therefore, 94% of the genes were similarly affected and 6% in opposite direction. This contrasts to a similar study in rice, where 50% of the genes were affected in opposite direction by ABA and ACC^15^.

The second objective of this study was to identify marker genes for the early influences of ABA, ACC, Epi, Me-JA and SA hormone treatments in potato. Most of the marker genes commonly used for hormone synthesis and signalling pathways are derived from *A. thaliana*. Marker genes for ABA response include *ABI* genes in *A. thaliana* and genes containing the ABA-responsive elements ABRE^9^,^10^,^28^. In this study in potato, an ABRE binding factor (DMG400008011) was uniquely 30-fold up-regulated by ABA at both 1 h and 6 h after treatment (Table S1). In addition to the ABRE binding factor, a transcript encoding Homeodomain 20 transcription factor (DMG400000248) was specifically induced by ABA and displayed approximately 30-fold induction at 1 h after treatment and 80-fold induction at 6 h in the microarray (Fig. 3).

Marker genes for ET signalling in *A. thaliana* encode ETHYLENE INSENSITIVE 3 (EIN3) and EIN3-like transcription factors (EIL) that further regulate other marker genes like ETHYLENE RESPONSE FACTOR 1 (ERF1) and ETHYLENE RESPONSE DNA BINDING FACTORs 1 to 4 (EDF1 – EDF4)^14^,^29^,^30^,^31^. In this study, the potato homolog of EIN3 (DMG400029908), whilst detectable, was not differentially expressed. The expression of the ERF1 homolog in potato (DMG400013402) remained below the limit of detection (Table S2). However, a suitable alternative marker gene, osmotin OSML15 (DMG400003057), was identified that displayed strong (up to 80-fold) and specific induction at 6 h (Fig. 3).

The three most commonly used marker genes for BR in *A. thaliana* are DWARF4 (DWF4) and CONSTITUTIVE PHOTOMORPHOGENIC DWARF (CPD), both of which are down-regulated by application of BR^32^, and EXPANSIN8 (EXP8) which is induced by BR treatment^33^. The closest potato homologs to DWF4 and CPD are annotated as cytochrome P450 genes (DMG400014902 and DMG400023819, respectively). Both genes were expressed although expression of the DWF4 homolog was close to the detection limit, but neither of the genes was differentially affected by Epi treatment. An expansin (DMG400016650) is the potato homolog to AtEXP8 but the raw expression values were below the confidence threshold for detection (Table S2). Genes involved in BR signalling are the leucine-rich repeat receptor-like kinase BRASSINOSTEROID-INSENSITIVE 1 (BRI1) and BRI1-ASSOCIATED KINASE 1 (BAK1)^13^,^34^,^35^,^36^. The potato BRI1 gene (DMG400019698) was detectable, although not differentially expressed following Epi application at 1 h after treatment, but not expressed at 6 h (Table S2). Similarly, expression of the BAK1 potato homolog (DMG400012594) was detectable but no differential regulation was apparent (Table S2). Nevertheless, a gene annotated as auxin-induced SAUR gene (DMG400016561) was significantly more highly expressed in Epi treated plants at 6 h than in any other hormone treated plants (p-value ≤ 0.05; Fig. 3). Up-regulation of auxin-induced SAUR genes by treatment with brassinolides had been demonstrated previously^37^.

Typical JA marker genes encode JASMONATE ZIM domain (JAZ) proteins that, in the absence of jasmonate, repress transcription of jasmonate-inducible genes by binding to their promoters^14^,^38^. The JA signalling pathway has two major branches, the MYC and the ERF branch and JA-responsive marker genes for these branches encode VEGETATIVE STORAGE PROTEIN 2 (VSP2) and PLANT DEFENSIN 1.2 (PDF1.2), respectively^5^,^39^,^40^. Application of JA also leads to activation of APETALA 2/ETHYLENE RESPONSE FACTOR (AP2/ERF) transcription factors, which regulate the transcription of JA-responsive defence genes^41^,^42^. In this study, we also identified a jasmonate ZIM-domain protein 1 (DMG400002930) that was uniquely 7-fold up-regulated by Me-JA at 1 h after treatment and uniquely 4-fold up-regulated 6 h after treatment. The JA-specific expression was confirmed by qRT-PCR (Fig. 3). The potato homolog of the VSP2 gene (DMG400016494) was expressed but did not reveal differential expression at 1 h or 6 h post treatments in this experiment, whereas the raw expression values of the potato homolog to PDF1.2 (DMG400008516) were below the confidence threshold for detection (Table S2).

Typical *A. thaliana* SA marker genes encode PHENYLALANINE AMMONIA LYASE (PAL) involved in SA synthesis and PATHOGENESIS-RELATED (PR) genes involved in SA defence signalling^5^,^14^,^43^,^44^. The regulatory protein NONEXPRESSOR OF PR GENES1 (NPR1) plays a role in both JA and SA signalling because it is activated by SA and supresses JA responses^5^,^14^,^45^. In this study, the potato PAL1 homolog (DMG400031457) was expressed but was not significantly affected by any treatment (Table S2). The expression levels of the potato gene homolog of PR1 (DMG400005112) and of NPR1 (DMG401000923) were below the confidence threshold of detection (Table S2). A gene similar to *AtWRKY40* (DMG402007388), however, responded specifically to SA treatment (Fig. 3). The involvement of WRKY transcription factors in SA signalling in *A. thaliana* had been shown before^5^,^42^, but involvement of WRKYs is more complex in defence responses in rice^17^.

This study has provided novel insight into hormone signalling pathways in potato and represents a state-of-the-art reference database for the transcriptional regulation of genes by the major defence hormones. The hormones in our study did not specifically influence all the traditionally used hormone marker genes from *A. thaliana*. We therefore used the data presented in this study to develop new marker genes for the different plant hormones in potato. These newly identified marker genes will, in the future, be useful to ascertain the mode-of-action of novel elicitor compounds or to elucidate functions of pathogen effectors. In addition, the data generated in this study have been used to generate a new potato gene expression database which is also linked to the Spud DB Genome Browser to enable more comprehensive analysis. The potato gene expression database will accommodate future gene expression studies and will allow users to quickly determine expression changes for current potato gene models.

## Material and Methods

### Plant material, growth conditions and experimental setup

Single stem potato plants (*Solanum tuberosum* cultivar Desiree) were grown from tubers in individual pots in a glasshouse set at 18 °C for 16 h day length supplemented with artificial lighting and 14 °C for 8 h in the dark. Plants were grown for five weeks prior to treatments to obtain leaves from comparable developmental stages and maturity^46^. Plants were treated with 1 mM abscisic acid (ABA), 1 mM aminocyclopropanecarboxylic acid (ACC), 0.05 mM epibrassinolide (Epi), 1 mM methyl jasmonate (Me-JA) or 1 mM salicylic acid (SA). A water control was included. All solutions contained 10 μl DMSO ml^-1^ and all chemicals were obtained from Sigma Aldrich. For each treatment, six plants were sprayed with a total of 100 ml per hormone solution until runoff. After 1 h and 6 h post treatments, six compound leaves from the middle part of three plants per time point each were harvested and snap frozen in liquid nitrogen. Three independent biological replicates were conducted.

### RNA extraction

Total RNA was extracted from 100 mg of frozen ground leaf material using 0.5 ml of TRIzol Reagent and 0.2 ml of chloroform according to the manufacturer’s protocol. RNA was further purified using a phenol-chloroform step at pH 4.3 to eliminate DNA carryover. RNA concentration and quality (260/280 nm > 2.0) were measured using a Nanodrop 2000 UV-Vis spectrophotometer (Thermo Scientific). The integrity of the samples was checked using a Bioanalyzer (Agilent Technologies).

### Microarray analysis

High-throughput gene expression analysis of three independent biological replicates was performed using custom Agilent microarrays designed to represent predicted transcripts of assembly version 3.4 of the potato DM genome^19^: A-MEXP-2272 was designed to multiple transcript isoforms (53, 000 in total). Microarray experimental design and datasets are available in ArrayExpress (ebi.ac.uk/arrayexpress/; accession E-MTAB-3542). All microarrays were processed essentially as described by Hancock *et al.*^47^, with the following exception: a single-channel design was used throughout, with all RNA samples labelled individually with Cy3 as recommended (One-Color Microarray-Based Gene Expression Analysis protocol v.6.5; Agilent). All other hybridisation, washing and scanning procedures, along with feature extraction (FE) and microarray data normalisation in GeneSpring (v. 7.3; Agilent) were as described. Unreliable data flagged as absent in all replicate samples by the FE software were discarded. Statistical filtering of data was performed for each time point separately (1 h, 6 h) using analysis of variance (ANOVA; p-value ≤ 0.05) with Benjamini & Hochberg multiple testing correction. Volcano plots on the ANOVA-filtered gene-lists were used to identify significantly changing probes between control and each hormone treatment (≥2x fold-change; p-value ≤ 0.05). In some cases, different probes on the array are representative of the same gene. For consistency, gene names (PGSC0003DMG numbers abbreviated to DMG4XXXXXXXX) are given throughout the entire manuscript. For the identification of housekeeping genes that are suitable for downstream qRT-PCR validation, only genes that were consistently detected in all samples and displayed a normalised expression ratio between 0.8 and 1.2 were considered.

### Gene ontology analysis

For gene ontology classification, Blast2GO (version 2.8)^48^ was used to annotate the gene transcripts. For each treatment all overrepresented gene ontology (GO) terms (biological process, level 2) were analysed.

### Marker gene analysis

Previously described *A. thaliana* genes involved in hormone signalling or described as phytohormone markers were assessed for their gene expression profiles and specificity in potato (Table S2). Protein sequences from the *A. thaliana* marker genes were blasted against potato proteins using the Spud DB website (http://potato.plantbiology.msu.edu). The DMG numbers corresponding to the potato proteins (DMPs) were used to identify the corresponding genes on the microarray^49^. Where readily identifiable, the reciprocal best hits (ERF1, DWF4, CPD, EXP8, BRI1, BAK1, PAL1) were selected or, if not discernible, the top potato protein blast hits (EIN3, VSP2, PDF1.2, PR1, NPR1) were utilised.

### Expression validation via qRT-PCR

For gene expression validation, cDNA was synthesised from 2 μg of total RNA from hormone treated leaf tissue using Ready-To-Go You-Prime First-Strand Beads (GE Healthcare Life Sciences) in conjunction with oligodT and random hexamer primers mixed in a 1:1 ratio (Thermo Fisher Scientific). Amplification of six target and two housekeeping genes (StNuclear [DMG400023981 a small nuclear ribonucleoprotein G] and St40S [DMG400020803]) was assessed (Table 2). All reactions were setup in a total volume of 25 μl using 2x FastStart Universal Probe Master (Rox) (Roche Diagnostics Corporation) and Universal ProbeLibrary probes (Roche Diagnostics Corporation) as specified by the manufacturer. Primer and probe combinations are listed in Table 2. A StepOnePlus™ Real-Time PCR System (Life Technologies) was used with the following conditions for all genes: initial denaturation for 10 min at 95 °C followed by 40 cycles of 15 s at 95 °C and 60 s at 60 °C. Relative expression of the target genes was calculated using a modified Pfaffl method^50^ with the geometric mean of the two housekeeping genes as the reference.

## Additional Information

**How to cite this article**: Wiesel, L. *et al.* A transcriptional reference map of defence hormone responses in potato. *Sci. Rep.* **5**, 15229; doi: 10.1038/srep15229 (2015).

## Supplementary Material

Supplementary Table S1

Supplementary Table S2

Supplementary Figure S1

## Figures and Tables

**Table 1:**
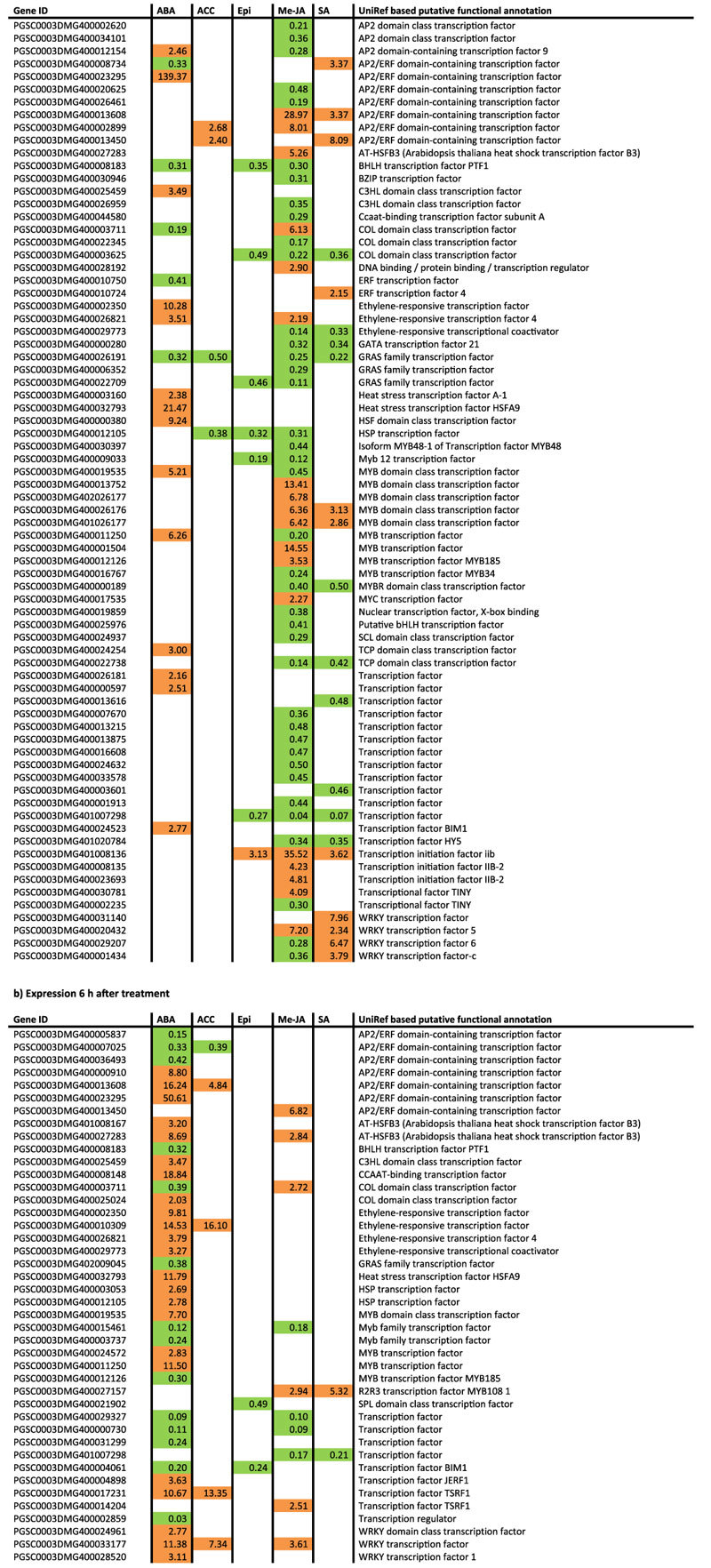
Transcription factors (UniRef annotation) that are differentially expressed at 1 h (Table 1a) and 6 h (Table 1b) after hormone treatments are shown.

Values represent fold changes in gene expression in hormone treated plants in comparison to control plants. Up-regulated genes are highlighted in orange, down-regulated genes are highlighted in green. ABA (abscisic acid); ACC (aminocyclopropanecarboxylic acid); Epi (epibrassinolide); Me-JA (methyl jasmonate); SA (salicylic acid).

**Table 2:** Primers and Universal Probe Library identities used for the amplification of two housekeeping genes (StNuclear and St40S) and six target genes (StHB20, StOsmotin2, StSAUR2, StJas, StmRNA, StLEDI).

Name	Accession	UPL probe	Left primer	Right primer
StNuclear	DMG400023981	#114	tggtgacaattgaagcgttg	tggggcataaacaaagatcc
St40S	DMG400020803	#69	gccactggtggcaagaag	ctggcggccaagttcata
StHB20	DMG400000248	#143	gctgcttcagcagtctgtca	acctcctcgcgttgttattg
StOsmotin2	DMG400003057	#10	ctgcccctacaccgtttg	caccaactctgacctctctcg
StSAUR2	DMG400016561	#150	cacaaagcattgctcctatcaa	tgttggtttccactttcttgg
StJas	DMG400002930	#145	ctcaacaaacagctaccaccac	cgatgaatcacttgatttctcaat
StmRNA	DMG402007388	#133	aaaatatggtcaaaaagtgacaagag	catgttggtgcaaatgaacac
StLEDI	DMG402018777	#122	ggatgaaaacagttggggtaaa	ccttcctcatgggtacaagg

StNuclear is annotated in potato as a small nuclear ribonucleoprotein G and the closest homolog in *A. thaliana* is AT2G23930.1 with the same annotation. ST40S, annotated as a 40 S ribosomal protein S8 in potato, has similarity to *A. thaliana* AT5G59240.1, also a ribosomal protein S8e family protein. The closes *A. thaliana* homolog to potato StHB20, a homedomain 20 transcription factor, is AT3G61890.1 encoding for homeobox 12. StOsmotin2 has been annotated as Osmotin OSML15 in potato and the highest sequence identity in *A. thaliana* is to AT4G11650.1, annotated as osmotin 34. StSAUR2, an auxin-induced SAUR, shows homology to *A. thaliana* gene AT1G29510.1, a SAUR-like auxin-responsive protein. StJas, annotated as jasmonate ZIM-domain protein 1 in potato, displays homology to AT1G19180.1 with the same annotation. StmRNA is a 1346 bp sequence with no further annotation in potato with the highest sequence similarity to *A. thaliana* AT1G80840.1, a WRKY DNA-binding protein 40. StLEDI is annotated as LEDI-5c with the highest sequence similarity to AT1G76690.1, encoding for 2-oxophytodienoate reductase 2.
