# Supplementary material for: A transcriptional reference map of defence hormone responses in potato
Source: Sci Rep. 2015 Oct 19;5:15229. doi: 10.1038/srep15229 (PMC4610000; doi:10.1038/srep15229)
Supplement: Supplementary Figure S1 [file srep15229-s3.pdf]

A transcriptional reference map of defence hormone responses in potato

Lea Wiesel[1], Jayne L. Davis[1], Linda Milne[1], Vanesa Redondo Fernandez[1], Miriam B. Herold[1], Jill Middlefell Williams[1], Jenny Morris[1], Pete E. Hedley[1], Brian Harrower[1], Adrian C. Newton[1], Paul R. J. Birch[2], Eleanor M. Gilroy[1], Ingo Hein[1]

[1] The James Hutton Institute, Invergowrie, Dundee, DD2 5DA, UK
[2] The Division of Plant Sciences, College of Life Science, University of Dundee at the James Hutton Institute, Dundee, UK

*Correspondence:
Ingo Hein
The James Hutton Institute
Invergowrie
Dundee
DD2 5DA
UK
Ingo.Hein@hutton.ac.uk
+44 (0)1382 568869

Figure S1: Screenshots of the potato gene expression database (http://ics.hutton.ac.uk/solarray) to illustrate the layout and information accessible via the database.

The James Hutton Institute

Transcript

DMT400000694

- Source Contig **ST4.03ch01**
- Source Gene **DMG400000248**

Available Data

|Microarray Probes|
|Spudb GBrowse|
|Homologues|
|DNA Sequence|

solarray

- solarray Home
- BLAST Search
- Keyword Search
- Samples and Analysis

Links

- James Hutton Institute Home
- BioSS Home

Microarray Probes on PGSC0003DMT400000694

Probe ID	Chip	Probe Sequence
CUST_3228_PI426222305	JHI_St_60k_v1	GGAGGGTTGATGTTGTTGCAACGAGTTTGAGTTTAAATTTTATGCACGATGTAGATAATA

Spudb GBrowse for Transcript PGSC0003DMT400000694

View details at PGSC Spudb GBrowse

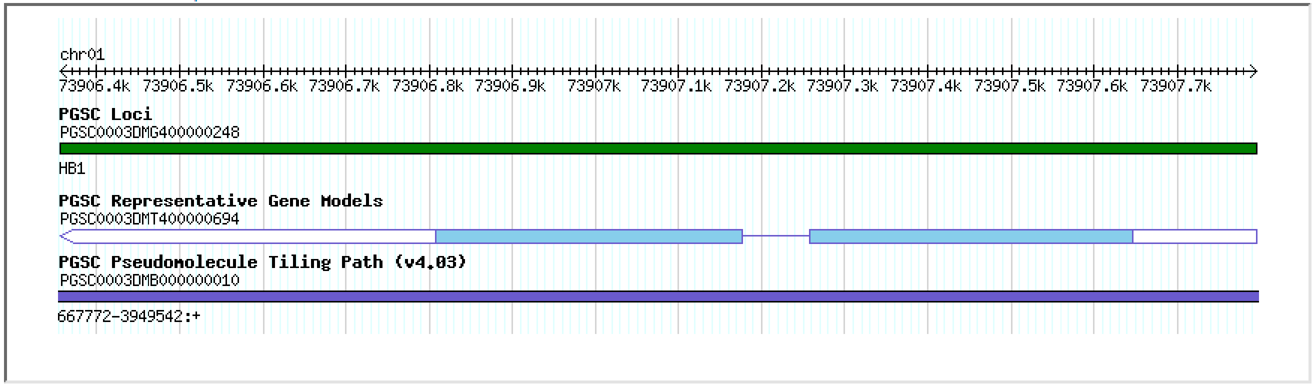

Source Transcript PGSC0003DMT400000694 - Homology to Model Species (BLASTX to E-value < 1e-50)

Database	Link to BLAST Hit	Frame	E-value	Score	% Identity	Description
Tomato (ITAG)	None	-	-	-	-	-
TAIR PP10	AT3G61890.1	+3	2e-64	210	114/219 (52%)	homeobox 12 | chr3:22914346-22915239 REVERSE LENGTH=235

Transcript Sequence (1359 bp)

>PGSC0003DMT400000694 1359 pgsc_dm_v3.4
GTTACAAGTTAAAGAATTCCCCACCAGAGCTGAACACCATAAAGATACCATTCTATCAAC

The James Hutton Institute

Source Transcript

DMT400000694

Source Contig
ST4.03ch01
Source Gene
DMG400000248

Available Data

|Microarray Probes|
|Microarray Signals|
|Homologues|
|Spud DB GBrowse|

solarray

solarray Home
BLAST Search
Keyword Search
Samples and Analysis

Links

James Hutton Institute Home
BioSS Home

Probe ID	Chip name	Transcript ID	Probe Sequence
CUST_3228_PI426222305	JHI_St_60k_v1	DMT400000694	GGAGGGTTGATGTTGTTGCAACGAGTTTGAGTTTAAATTTTATGCACGATGTAGATAATA

All Microarray Probes Designed to Gene DMG400000248

Probe ID	Chip name	Transcript ID	Probe Sequence
CUST_3228_PI426222305	JHI_St_60k_v1	DMT400000694	GGAGGGTTGATGTTGTTGCAACGAGTTTGAGTTTAAATTTTATGCACGATGTAGATAATA

Microarray Signals from CUST_3228_PI426222305

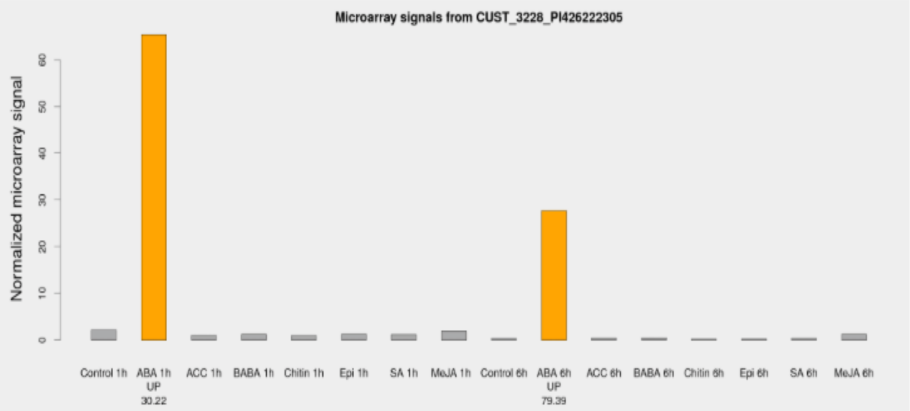

Key: Orange = UP–regulated, Green = DOWN–regulated. The fold change values (UP >= 2, DOWN <= 0.5) are noted under labels.
Only significant fold changes (treatment vs. control) are highlighted.

Treatment	Raw signal	Raw Std Err	Normalized signal	Normalized Std Err
Control 1h	136.816	9.85226	2.16464	0.301652
ABA 1h	3702.33	472.377	65.4119	6.91331
ACC 1h	78.4187	33.517	0.988557	0.455013
BABA 1h	77.1449	13.6081	1.23365	0.130704
Chitin 1h	75.5278	34.7612	0.961735	0.825268
Epi 1h	81.6501	27.5061	1.25912	0.659514
SA 1h	77.1636	6.66213	1.18517	0.14662
Me-JA 1h	99.6506	10.6408	1.91716	0.131227
Control 6h	27.1781	10.0001	0.349301	0.167193
ABA 6h	1896.31	269.064	27.7307	3.2938
ACC 6h	31.8264	4.71579	0.440542	0.0640434
BABA 6h	37.4245	4.48784	0.525531	0.0645788
Chitin 6h	15.0036	3.99104	0.216241	0.0631077
Epi 6h	19.5626	8.70142	0.227878	0.0901232
SA 6h	23.789	4.01112	0.377099	0.0664288
Me-JA 6h	79.5084	16.7325	1.21923	0.360543

Table S1: All probes significantly affected by application of any of the five hormone treatments at 1 h and 6 h after treatment (p-value ≤ 0.05). Shown are fold changes in comparison to the control treatment, normalised and raw values including standard error and further information on each probe including the gene annotation.

Table S2: Normalised and raw expression values including standard error of potato homologs of previously described *A. thaliana* hormone markers. The marker specificity, *Arabidopsis* gene name and ID are shown alongside the corresponding potato gene (gene, transcript and protein) and their normalised and raw values at 1 and 6 hours post hormone treatments. Where readily identifiable, the reciprocal best hits (RBH) were selected or, if not discernible, the top potato protein blast hits (TH) were utilised.
